# Mapping Heart Development in Flies: Src42A Acts Non-Autonomously to Promote Heart Tube Formation in *Drosophila*

**DOI:** 10.3390/vetsci4020023

**Published:** 2017-04-24

**Authors:** Jessica Vanderploeg, J. Roger Jacobs

**Affiliations:** 1 Department of Biology, Taylor University, 236 West Reade Ave., Upland, IN 46989, USA; jessica_vanderploeg@taylor.edu; 2 Department of Biology, McMaster University, 1280 Main St. W., Hamilton, ON L8S 4K1, Canada

**Keywords:** heart, *Drosophila*, model organism, Integrin, Src42A, FAK, genetics, amnioserosa

## Abstract

Congenital heart defects, clinically identified in both small and large animals, are multifactorial and complex. Although heritable factors are known to have a role in cardiovascular disease, the full genetic aetiology remains unclear. Model organism research has proven valuable in providing a deeper understanding of the essential factors in heart development. For example, mouse knock-out studies reveal a role for the Integrin adhesion receptor in cardiac tissue. Recent research in *Drosophila melanogaster* (the fruit fly), a powerful experimental model, has demonstrated that the link between the extracellular matrix and the cell, mediated by Integrins, is required for multiple aspects of cardiogenesis. Here we test the hypothesis that Integrins signal to the heart cells through Src42A kinase. Using the powerful genetics and cell biology analysis possible in Drosophila, we demonstrate that Src42A acts in early events of heart tube development. Careful examination of mutant heart tissue and genetic interaction data suggests that Src42A’s role is independent of Integrin and the Integrin-related Focal Adhesion Kinase. Rather, Src42A acts non-autonomously by promoting programmed cell death of the amnioserosa, a transient tissue that neighbors the developing heart.

## 1. Introduction

A properly functioning heart is intimately linked to its structure. Defects in this structure may trigger immediate or later-onset heart disease. Congenital heart defects (CHDs) such as patent ductus arteriosus, atrial or ventricular septal defect, and tetralogy of Fallot, have been noted for both large and small animals, including cats [1], dogs [2], horses [3], and cows [4,5]. Development of the heart can effectively be studied at the genetic level to reveal the genes that determine the identity of heart cells and that dictate the response of heart progenitors to signals from neighboring cells and tissues. Clinical observations, especially those which involve domestic purebreds, suggest that the underlying cause of CHDs is often heritable [6,7]. This is supported by studies in the last fifteen years which have begun to link specific CHD’s to genetic aetiologies [8,9]. However, despite the increasing recognition of heritable factors, a full understanding of the genetic aetiology is hindered by the complex and multifactorial nature of these diseases. Even in instances where a specific genetic factor has been correlated to heart development, it remains difficult to definitively establish a causal relationship and determine how this genetic abnormality contributes to disease [10]. The underlying cellular or molecular mechanism remains a mystery.

While few genes have been correlated to CHDs in small and large animals in the clinic, experimental studies in mice have been used to ask targeted questions about likely candidates. Insights from genetic evidence in humans suggest a link between cardiovascular disease and integrin-related factors [10,11,12]. The Integrin heterodimer is a protein adhesion complex that spans the cellular membrane and connects the extracellular matrix to the intracellular cytoskeleton and various signaling cascades. Multiple research groups have studied knock-out mice lacking the alpha (α) or beta (β) subunits of the Integrin receptor. In mice, the β1 subunit is broadly expressed during development and serves as the β-integrin subunit for about half of all known vertebrate heterodimers (reviewed in [13]). Thus, it is not surprising that β1-null mice die very early in development prior to embryo implantation (at E5.5; [14,15]). However, tissue-specific loss of β1 reveals roles for development of multiple organs, including the heart [16,17,18]. Knock-out mice for α4 or α5, two of the β1 partners, also exhibit early embryonic lethality (by E8-10; [19,20,21]), with prominent defects in the epicardium of the heart (α4) and reduced blood vessel integrity (α4 and α5; [20,21]). 

While vertebrate models have correlated specific factors like Integrin to CHDs, cell culture and simpler genetic models are powerful tools to map the complex signaling networks surrounding these genes. In particular, here we highlight the fruit fly (*Drosophila melanogaster*), which has become a well-studied model for cardiovascular development and aging for experimental biologists (reviewed in [22]). Although far simpler anatomically, development of the *Drosophila* embryonic heart is reminiscent of early vertebrate heart morphogenesis [23,24]. Development of both organs requires specification of the heart cells, medial alignment of two parallel rows of cells, medial-to-midline migration of these cells, and organization and rearrangement of these midline cells into a lumen-containing heart organ (reviewed in [25]). *Drosophila* is the only well-studied invertebrate model system with a heart comparable to vertebrates. Thus, it is a unique system, valuable because of its simplicity and the powerful genetic and proteomic manipulations and analysis possible (reviewed by [23,26]). The tools utilized in this study include mutant alleles, tissue specific inhibition or overexpression of target genes, labeling and imaging of specific cell types at the single-cell level, and non-invasive live imaging of developing embryos using proteins tagged with fluorescent markers. While alleles or specific mutations in genes may be correlatively linked to heart and vascular diseases in veterinarian animals, it is through the tools employed in model organism research that normal function of these genes can be established and the etiology of the correlated diseases understood.

Consistent with the correlative evidence found in mice studies, recent research in *Drosophila* has illustrated an essential role for Integrins in heart development [27]. We demonstrated that Integrins are involved in multiple steps of the process including heart cell migration, cardioblast cells (CB) polarization, and assembly of the heart tube [27]. However, Integrin signaling is complex (reviewed in [28]), and it remains less clear how Integrins guide the heart cells to trigger cardial cell migration and tube formation. Using a combination of genetic and cell biological *Drosophila* techniques, our lab has performed a targeted approach to map the extracellular and intracellular protein network involved in integrin-mediated heart development. This approach has thus far revealed a critical role for the intracellular signaling adaptor Talin [29] and extracellular matrix regulation [30]. Here we extend our study to explore a possible Integrin-dependent role for Src42A, one of two *Drosophila* Src kinases. Src is a non-receptor tyrosine kinase that transmits cell signals in a variety of contexts, including cell migration [31,32]. Activated Src is recruited to Integrin-tethered focal adhesions [33,34] where it phosphorylates several Integrin complex proteins including FAK and Paxillin [31,35,36]. When examined in cardiomyocytes, in vitro, Src signals participate in mechanical stress signaling that can trigger cardiac hypertrophy [34,37]. Here we use the in vivo *Drosophila* heart model to test the hypothesis that Src42A transmits Integrin signals to the developing heart. Our experimental approach employs mutant analysis, genetic interaction tests, tissue-specific protein inhibition, and cell biological analysis of living and fixed tissue. Our studies suggest that Src42A acts non-autonomously during heart development by promoting programmed cell death of migration-inhibiting extraembryonic amnioserosa tissue.

## 2. Materials and Methods 

### 2.1. Drosophila Strains and Genetics

All fly strains and cross schemes were maintained according to standard conditions at 25 °C. All mutant alleles were maintained in a *w^1118^* background over a ftz > lacZ or en > lacZ marked balancer chromosome. The following mutant alleles were tested: *src42A*^E1^ (Bloomington *Drosophila* Stock Center, BL #6408), *src42A*^k10108^ (BL #10969), *src42A*^26-1^ [38], *fak56D*^KG00304^ (BL #13080), and rhea^1^ (BL #2296). For overexpression studies, UAS-Src42-YF [39] and UAS-Src42-DN [38] were crossed to dmefGal4 [40]. Yet1 flies express YFP in amnioserosa perimeter cells [41]. In live imaging experiments, dmefGal4, UAS-moesin-mCherry [42], and tup-GFP [43] were all present in a single copy in *src42A^E1^/src42A^E1^* embryos.

### 2.2. Immunohistochemistry

Embryo collection, fixation, and immunolabeling protocols were completed as previously described [29,44]. The following primary antibodies were used: rabbit anti-MEF (1:5000; [27]), chicken anti-βGal (1:150; [45]), mouse anti-Hindsight (1:30; Developmental Studies Hybridoma Bank), and chicken anti-GFP (1:1000; Cedarlane). Embryos prepared for light microscopy were treated with a biotinylated secondary antibody (1:150; Jackson ImmunoResearch), incubated with the avidin and biotinynlated peroxidase containing Vectastain ABC system (Vector Laboratories, Burlingame, USA), and reacted with 3,3- Diaminobenzidine Tetra hydrochloride (DAB, Gibco-BRL) and hydrogen peroxide. Images were acquired using a Zeiss Axioskop microscope (with a RETIGA 1300i digital camera, Qimaging, Surrey, Canada) and OpenLab software (PerkinElmer, Woodbridge Canada). For embryos prepared for fluorescent confocal microscopy, the secondary antibodies were Alexa 488 and 594 (Invitrogen). Fluorescent-labelled embryos were imaged on a Leica SP5 confocal microscope (Leica Microsystems, Buffalo Grove, USA). Frontal images displayed are maximum projections of 3–10 slice stacks taken 1 μm apart in the heart chamber. Images of embryo z-sections (transverse images) are single slice images of the heart chamber. All images were processed with ImageJ (Rasband, W.S, NIH, Bethesda USA) and figures were assembled using Adobe Photoshop CC 2015.5 (Adobe Systems Incorporated, San Jose, USA).

### 2.3. Preparation of Tissue Sections for Electron and Light Microscopy

Embryos were prepared as outlined previously [45]. Embryo sections were examined on a JEOL 1200EXII microscope (electron microscopy) or a Zeiss Axioskop microscope with a Qimaging RETIGA 1300i digital camera (light level microscopy).

### 2.4. Live Imaging of Embryos

Live imaging of embryos was performed using the hanging drop method [29,46]. Embryos were imaged on a Leica SP5 confocal microscope. Images are maximum projections of 20–25 slices taken 1 µm apart. 

## 3. Results

### 3.1. Key Steps in Drosophila Melanogaster Embryonic Heart Development

**Figure 1:**
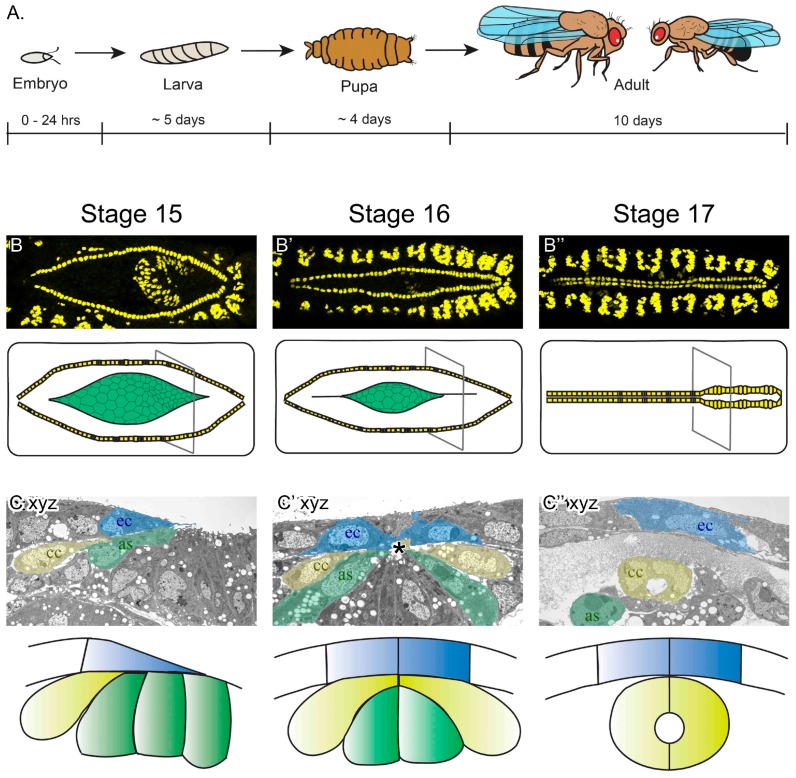
Drosophila melanogaster embryonic heart development. **(A)** Although the lifecycle of flies, from egg-laying to adult is approximately 10 days at 25 °C, embryogenesis completes in just 24 hours. It is during embryogenesis that the heart develops into a linear tubular organ. **(B–B’’)** Images of the dorsal surface of fixed embryos imaged with a confocal fluorescent microscope (top panels are labeled for dMEF, present in the nuclei of somatic and heart muscle). By embryonic stage 15, the heart cells (yellow in schematic) are aligned in two lateral rows, flanking the amnioserosa tissue (green in schematic). As embryogenesis continues, the cardioblast cells (CBs) migrate towards the dorsal midline (stage 15–17). By the end of stage 17, the embryonic heart is a mature vessel, capable of pumping hemolymph throughout the body. The slightly wider heart vessel is at the posterior (to the right), while the outflow tract extends anteriorly (to the left). **(C–C’’)** In transverse sections of fixed embryos imaged using an electron microscope, migrating cardial cells (yellow) follow the dorsal ectoderm leading edge (blue) and contact the amnioserosa (green) ventrally (stage 15). As CBs approach the midline, they migrate between the ectoderm and amnioserosa to reach their contralateral partner at the midline (asterisk, stage 16). Once at the midline, the CBs adopt a crescent-shape morphology and enclose the lumen (stage 17). At this stage, dorsal closure has completed; the ectoderm covers the dorsal surface of the embryo and the amnioserosa has invaginated and the cells are undergoing apoptosis. Dorsal is at the top of C–C’’ as, amnioserosa; ec, ectoderm; cc, cardial cells.

**Figure 2:**
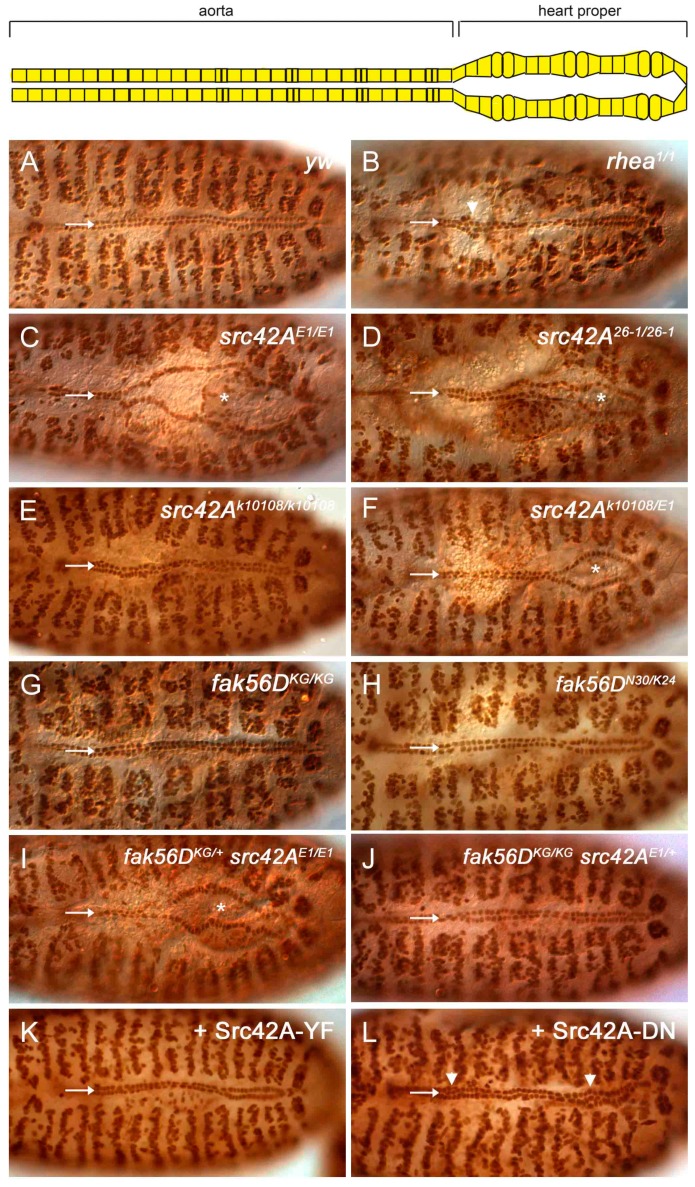
Loss of Src42A results in incomplete cardioblast migration. **(A)** By stage 17, the two CB rows are aligned along the dorsal midline in wildtype embryos. The two rows are close, but a narrow gap remains at the midline where the tube lumen is positioned (arrow). **(B)** In mutants of *rhea*, the gene encoding the Integrin-adaptor protein Talin, migration completes. However, occasional cell clumping occurs (arrowhead) and the space between the CBs rows is absent (arrow; [29]). **(C–F)** In *src42A^E1^* and *src42A^26-1^* null mutants, the posterior CBs fail to migrate to the midline (arrow) and the embryos exhibit an “open heart” phenotype (asterisks mark the open heart). The CBs in the *src42A^k10108^* hypomorph migrate fully to the midline (E), although an open heart phenotype is observed in some *src42A^E1^*/*src42A^k10108^* mutants (F, asterisks). **(G–J)** Similar to wildtype embryos (A), *fak56D* mutant embryos have normal CB alignment and migration (G, H). Reducing the gene copy of *fak56D* did not enhance the delay in CB migration in *src42A^E1^* mutant embryos (I) Reducing the gene copy of *src42A* did not induce a heart phenotype in *fak56D* mutant embryos (J). **(K)** Constitutively active Src42A was overexpressed in the heart and somatic muscles. Similar to wildtype, the heart cells were aligned, migrated to the midline, and maintained a narrow gap between the two rows. **(L)** Expression of a dominant negative form of Src42A kinase did not prevent CB migration. However, cells were frequently misplaced relative to neighboring cells, resulting in cell clumps (arrowhead). Posterior of the heart is to the right in all panels.

The Drosophila melanogaster life cycle completes in approximately ten days (Figure 1A). By the end of embryogenesis (24 h), the heart structure is formed and starts to beat. The fly embryonic heart is a linear tube, analogous to the vertebrate heart before looping morphogenesis (Figure 1B’’; see also Figure 2). It is comprised of two parallel rows of muscular cardioblast cells (CBs) that form the mature heart at the dorsal midline of the embryo. Development of the heart initiates during embryogenesis with the specification of two heart fields. These fields align laterally along the embryo (Figure 1B,C), adjacent to the amnioserosa, an extraembryonic cell layer that transiently covers the dorsal surface of the embryo. Another tissue layer, the ectoderm, covers the cardial cells dorsally (Figure 1C). Prior to the heart forming a tube, the two rows of CBs migrate collectively through the embryo and meet at the midline. In Drosophila, CB migration occurs simultaneously with dorsal closure, a process whereby the ectoderm migrates towards the dorsal midline and the transient amnioserosa cells are internalized and undergo apoptosis [47]. Following migration, an initial dorsal contact is made between contralateral CBs (Figure 1C’). The CBs fill the space vacated by the midline amnioserosa cells, immediately ventral to the ectoderm (Figure 1C’–C’’). The CBs become crescent-shaped and adhere ventrally to wrap around and fully enclose the inner lumen and complete the heart tube (Figure 1C’’). 

### 3.2. Src42A Is Required for Drosophila Heart Development

The requirement for specific factors in development can be tested experimentally by removing the factor and observing if development can still proceed normally. In *Drosophila*, a target protein can be removed by introducing a loss-of-function mutation in the gene that encodes that protein. To test if *Src42A* is required for *Drosophila* heart development, we compared the embryonic hearts of *src42A* mutants to wildtype and *rhea* mutants (*rhea* is the gene that encodes for Talin, an essential Integrin signaling adaptor; [29]). The heart was visualized by labeling the embryos with an antibody that binds to dMEF, a muscle-specific transcription factor. In late stage wildtype embryos, CB migration has completed and the cells are aligned along the dorsal midline (Figure 2A). In *rhea* mutants, migration completes, but no midline space remains between the CB rows, indicating that the heart is unable to form an open tube (Figure 2B, [29]). In contrast, embryos homozygous for the null *src42A^E1^* mutation frequently exhibited an “open heart” phenotype, in which posterior CBs failed to reach the midline (Figure 2C asterisks). This is analogous to a cardia bifida phenotype in mammals. To confirm this was not an allele-specific phenotype perhaps due to genetic abnormalities elsewhere in the genome, several independent *src42A* mutant alleles were tested. Embryos carrying a partial deletion of *src42A* (*src42A^26-1^;* [38]) had a similar open heart phenotype (Figure 2D). Embryos homozygous for a *src42A* hypomorphic allele had normal CB alignment (Figure 2E), however transheteroallelic embryos for hypomorphic and null *src42A* alleles occasionally displayed stalled or delayed posterior CB migration (Figure 2F). This suggests that Src42A is essential for full migration of the CBs, but that low levels of Src42A (as in the hypomorphic mutant) are sufficient for migration to complete. The difference in phenotypes between the *rhea* and *src42A* mutants suggests it is unlikely that Src42A is working together with or downstream of the Integrin-Talin signaling complex. 

In multiple vertebrate contexts, Src works downstream of Integrins with Focal Adhesion Kinase (FAK) to activate signal transduction [31,32,34]. In *Drosophila*, there is a single FAK encoded by the gene *fak56D*. Similar to vertebrate FAK, Fak56D has a focal adhesion targeting domain and a binding site for the SH2 domain of Src, but the function of its sequence-inferred predicted kinase domain remains unclear [48,49,50]. Thus, it is likely that some, but perhaps not all, vertebrate and fly FAK cellular functions are shared. One of the strengths of the *Drosophila* model is the ability to use genetics to map signaling pathways. For example, Tsai et al. studied the requirement for Fak56D and Src42A in the *Drosophila* nervous system [51]. Although flies heterozygous for *src42A^E1^* are healthy, Tsai et al. demonstrated that introducing a *src42A^E1^* mutation worsened the phenotype severity of a *fak56D* hypomorph in the nervous system. This genetic interaction suggests that Src42A and Fak56D work together in a signaling pathway during nervous system development. To test if *fak56D* similarly works with Src42A during heart development, we assessed flies with disrupted *fak56D*. *fak56D* hypomorphic and null *Drosophila* embryos have no obvious heart phenotype (Figure 2G,H). This is not surprising as flies of these genotypes are viable and healthy [51,52]. Therefore, to further test if Fak56D modulates Src signaling within the heart, *fak56D* gene dosage was reduced in *src42A^E1^* mutant embryos. Reduction of *fak56D* did not exacerbate the *src42A^E1^* phenotype (Figure 2I). Similarly, halving the gene dosage of *src42A* did not induce a phenotype in *fak56D* homozygous mutant embryos (Figure 2J). This data suggests that Src42A, but not Fak56D, is necessary for proper migration of the cardial cells in *Drosophila*.

### 3.3. Src42A Is Required for Cell Migratory Dynamics in the Posterior Heart

**Figure 3:**
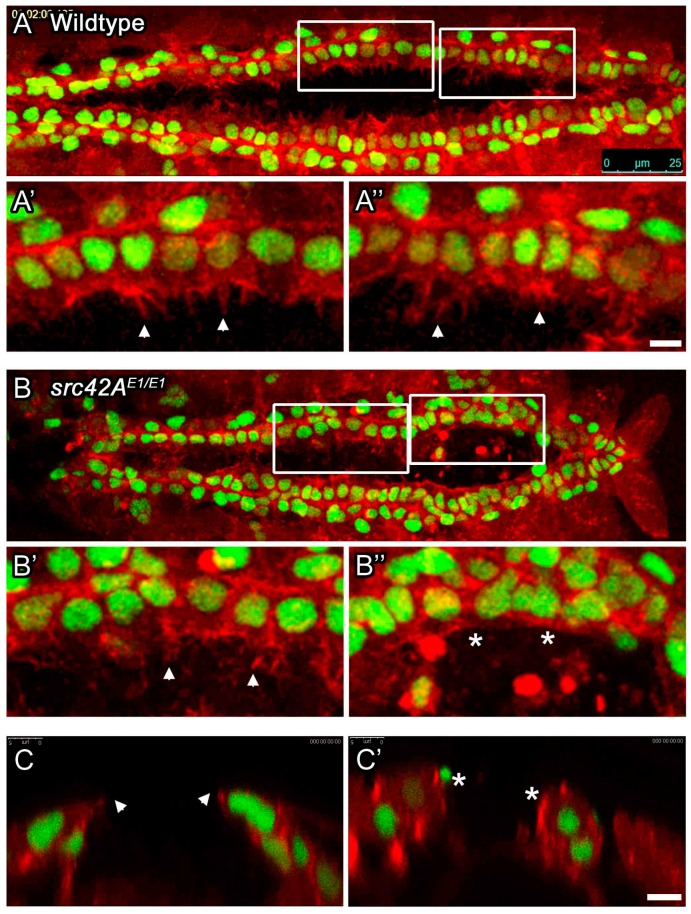
In *src42A* mutant embryos, cardioblasts with stalled migration lack leading edge membrane activity. The dynamic activity of the leading edge of migrating CBs was monitored in living embryos. Membrane actin was labeled with *dMEF-GAL4* regulated *UAS-moesin-mCherry* (red), while CBs were marked with *tup-GFP* (green). In wildtype embryos (**A**), both the anterior and posterior CBs extend highly dynamic processes towards the midline (arrowheads in **A’** and **A’’**). In *src42A^E1^* mutants, anterior CBs extend processes towards the midline (arrowhead in **B’**, cross-section in **C**), but posterior CBs remain rounded (**C’**) and do not extend such processes (asterisks in **B’’**, **C’**). Posterior of the heart is to the right (**A**, **B**); dorsal at top (**C**, **C’**). Calibration: 25 microns in A and B, 5 microns in all other panels.

Since our fixed-tissue studies demonstrated that CBs fail to reach the midline in *src42A* mutants, we wanted to explore how these mutants differ from wildtype during the cell migration stage. Migratory cells, whether in cell culture or live organisms, are characterized by a highly dynamic cell membrane that extends filopodia and lamelliopodia in the direction of migration [53,54]. In contrast to mammalian models, *Drosophila* is highly amenable to in vivo time-lapse analysis of heart development. In the *Drosophila* heart, CB “leading edge” membrane activity can be visualized in live embryos that express fluorescently tagged proteins. As the CBs migrate, they extend and retract numerous membrane protrusions, which increase as the CBs approach the midline [30]. Our studies on Integrin and Talin function in the heart demonstrated that these genes are required for the highly dynamic leading edge that migrating CBs extend [27,29]. Therefore, we reasoned that if Src42A is functioning downstream of Integrin to control CB migration, lack of Src42A should result in a similar loss of membrane leading edge activity. To directly test this, we compared the membrane activity in *src42A^E1^* mutants to wildtype. Consistent with previous studies, in wildtype embryos numerous membrane protrusions were observed across the length of the CB rows (Figure 3A–A’’). Interestingly, while a reduction in leading edge activity was observed in the posterior of the heart in *src42A^E1^* embryos (similar to *integrin* or *rhea* mutants), the CBs at the anterior of the heart retained a highly dynamic membrane activity similar to wildtype (Figure 3B—compare B’’ to B’). Transverse imaging revealed that CBs with high membrane activity extended a protrusion dorsally towards the midline (Figure 3C, arrows) while cells with minimal activity were rounded and did not extend dorsally (Figure 3C’). Thus, it appears that in contrast to *integrin* and *rhea* mutants [27,29], *src42A* nulls retain a highly dynamic leading edge in the anterior of the heart. In the posterior heart, however, CB leading edge activity is minimal, correlating with the inability of the posterior CBs to fully migrate to the midline.

### 3.4. Heart-Specific Modification of Src42A Function

While the data suggests that Src42A is required for heart development, it was unclear whether Src42A was required in the CBs themselves or if the mutant phenotype observed was due to disruptions in surrounding tissues (e.g., the amnioserosa or ectoderm). A powerful technique in *Drosophila* is the UAS-Gal4 system which allows temporal and spatial control of transgene expression (reviewed in [55]). Using this system, a constitutively active (YF) or wildtype version of Src42A was first overexpressed in the heart and other body-wall muscle tissue (Figure 2K). Following overexpression of either construct, the heart developed normally (Figure 2K and data not shown), suggesting that tight control of Src42A levels is not essential for heart development. To complement the Src42A overexpression expression, Src42A function was inhibited by expressing a dominant-negative Src42A (Src42A-DN). Src42A-DN lacks kinase activity [56] and ubiquitously expressing Src42A-DN during development results in late pupal lethality (this study). To directly test if the kinase activity of Src42A is required for cardial cell migration, Src42A-DN was expressed in the heart (using a muscle-specific enhancer). In these embryos, the heart cells fully migrated to the midline (Figure 2L), however the cells were frequently mis-positioned and formed small clumps (arrowheads in Figure 2L). This clumping phenotype suggests that Src42A may regulate adhesion between neighboring cells. Such a role would be consistent with a Src kinase-dependent E-cad trafficking mechanism [57,58]. However, the completed migration in the Src42A-DN embryos suggests that Src42A expressed within the heart itself does not regulate cardial cell migration. Another possibility is that Src42A is required within the heart cells for a kinase-independent role. Although Src42A-DN dominantly interferes with endogenous Src42A, it is unclear if it inhibits all Src function. While it interferes with the kinase activity of Src [56], it is possible that other protein-binding or “scaffolding” aspects of Src function remain intact and that these are sufficient for CB migration.

### 3.5. Delayed Migration in src42A Mutants Correlates with Stalled Internalization of the Amnioserosa

While the mutant data suggests that Src42A is required for heart development, it was intriguing that loss of Src42A only disrupted cardial cell migration in the posterior heart. Furthermore, the heart-specific expression of the Src42A-DN hints that the migration defects may not be due to Src42A signaling in the heart itself. We therefore hypothesized that the *src42A* mutant phenotype was due to disruptions in surrounding tissues (the amnioserosa or ectoderm). Previous studies have documented that Src42A is required for dorsal closure [38,59] and, consistent with this, a subset of *src42A* mutant embryos analyzed had incomplete dorsal closure (data not shown). Although somatic muscles positioned close to the midline suggested that dorsal closure was complete in at least some “open heart” *src42A* mutant embryos (e.g., Figure 1F), subtle phenotypes may not be readily apparent. 

**Figure 4:**
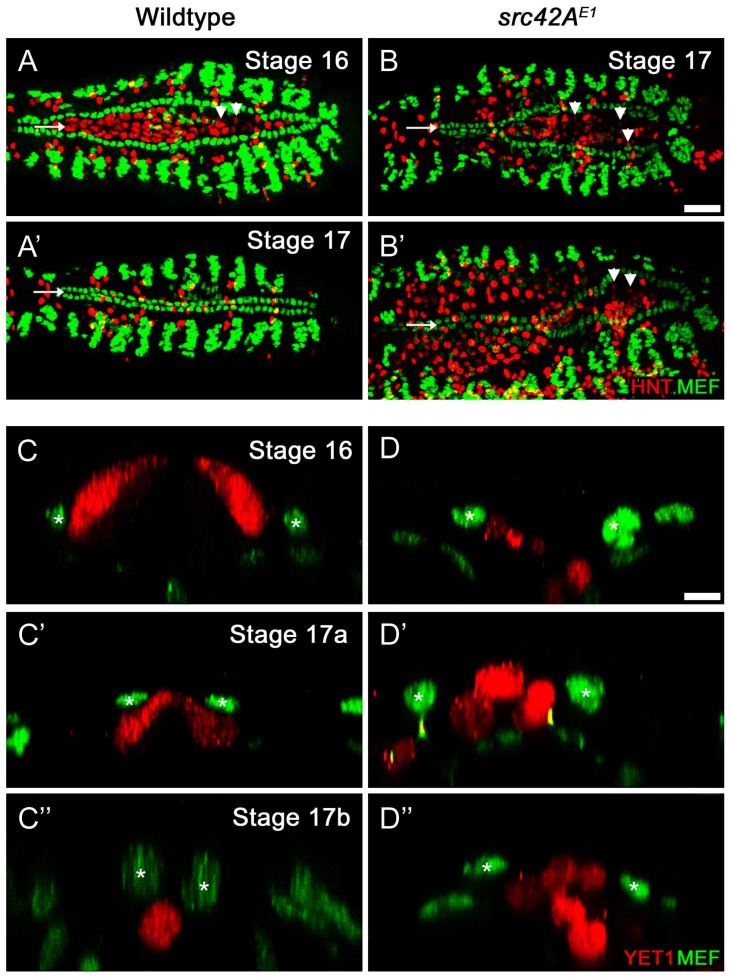
Amnioserosa cells remain at the midline in late stage *src42A^E1^* embryos. **(A–B’)** In wildtype embryos, the amnioserosa (red) is present at stage 16 (**A**) but has internalized and undergone apoptosis by the end of embryogenesis (stage 17, **A’**). In *src42A^E1^* mutants, amnioserosa cells persist in the posterior region of the embryo even at late stages (**B–B’**; arrowheads identify example amnioserosa cells; brightly red labeled cells in left of **B’** are part of the midgut). The nuclei of amnioserosa cells are labeled with αHindsight (HNT, red). The CB nuclei (and somatic muscle nuclei) are labeled with αMEF (green). Posterior of the heart is to the right. Calibration: 25 microns. **(C–D’’)** Transverse sections through the heart and amnioserosa tissue of wildtype and *src42A^E1^* mutant embryos. In wildtype embryos, migrating CBs (green, asterisks) move dorsally and eventually over the amnioserosa cells (red; **C–C’’**). Following migration, no amnioserosa cells are between the CBs; any remaining cells have been internalized (**C’’**). In contrast, late stage *src42A^E1^* embryos are characterized by persistent amnioserosa cells at the midline (**D–D’’**). Despite having the rounded shape characteristic of cells undergoing apoptosis, the amnioserosa cells do not internalize, but remain along the midline where the heart tube usually forms. CB nuclei (and flanking muscle nuclei in **C’** and **C’’**) are labeled with αMEF (green, asterisks), while YET1 marks the outermost amnioserosa cells (red). Dorsal is at the top. Calibration: 5 microns.

Therefore, to test if the CB migration delay was due to disruptions in the amnioserosa, we labeled the amnioserosa using an antibody against Hindsight (Hnt), which is present in the nuclei of the amnioserosa and midgut cells (Figure 4A–B’), and the enhancer trap YET1, which expresses Yellow Fluorescent Protein in the amnioserosa perimeter cells (Figure 4C–D’’). In wildtype embryos, the amnioserosa is visible at stage 16 (Figure 4A), but has internalized and undergone apoptosis by stage 17 (Figure 4A’). Already in stage 16 embryos, there are few amnioserosa cells remaining near the posterior region of the heart. However, in *src42A^E1^* embryos, the amnioserosa cells persist in the posterior region of the embryo, where the CB migration delay is observed (Figure 4B–B’). 

Transverse images of wildtype embryos shows that as CB migration proceeds, the CBs move dorsally and eventually over the amnioserosa cells (Figure 4C–C’’). Once CB migration is complete, no amnioserosa cells are between the CBs; any remaining cells have internalized into the embryo and appear round, characteristic of cells entering apoptosis (Figure 4C’’; [60]). This is not the case in *src42A^E1^* embryos: although the amnioserosa cells are rounded suggesting they are apoptotic, they persist at the dorsal midline (Figure 4D–D’’). Thus, it appears that the incomplete amnioserosa internalization is a physical barrier to CB migration and the migration defects observed in *src42A* mutants are likely not be the result of a requirement of Src42A in the heart itself.

### 3.6. Src42A Is Not Essential for Development of A Lumen

**Figure 5:**
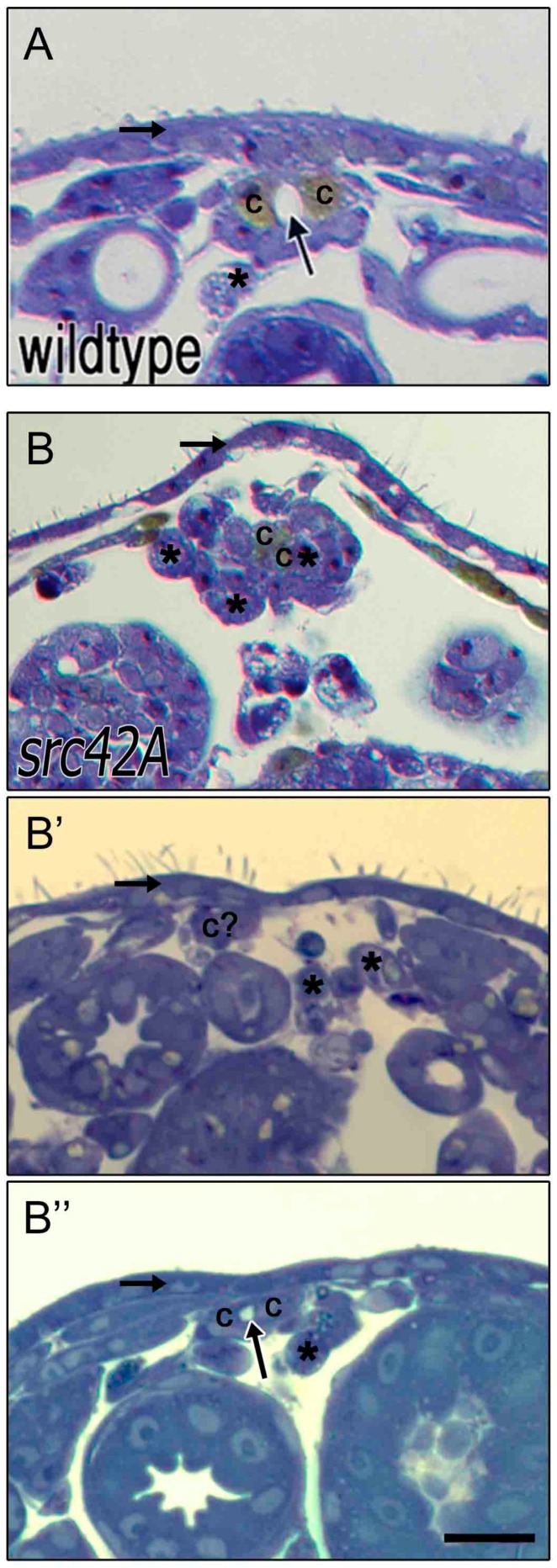
Cardioblasts at the midline are capable of developing an open lumen between contralateral cells. In stage 17 wildtype embryos **(A)**, contralateral CBs (“c”) are present at the dorsal midline and enclose a lumen (arrow). Dorsal to the heart is a thick and continuous ectoderm (horizontal arrow) and few if any amnioserosa cells remain ventrally (asterisks). In *src42A^E1^* mutants, the ectoderm layer is thin and variable (horizontal arrow in **B–B’’**) and amnioserosa cells persist at or near the midline (asterisks; for some cells marked with an asterisks, identity is uncertain). The CBs are not consistently present at the midline (e.g., c? in **B’**); however, when two contralateral CBs are present, a small lumen often forms (arrow in **B’’**). Dorsal at top. Calibration: 10 microns.

In *integrin* and *rhea* mutants, disruptions in cell migration and leading edge activity is correlated with the inability of the CBs to form a lumen [27,29]. If Src42A is required in the heart itself, we would expect that *src42A* mutants would form no lumen between CB cells that were able to reach the midline. However, if the migration delays are due to disruptions in amnioserosa internalization, contralateral CBs that meet at the midline would be able to form a lumen. Light level histology of embryo cross-sections was used to test these hypotheses. In late stage wildtype embryos, a lumen is visible between contralateral CBs (Figure 5A, see also Figure 1C’’). Dorsal closure has completed, as evidenced by the thick and continuous ectoderm layer dorsal to the heart (horizontal arrow). Any remaining amnioserosa cells have been internalized and are situated ventral to the heart (asterisks). In *src42A^E1^* embryos, there are a range of phenotypes. Consistent with the images described earlier (Figure 4D–D’’), some embryos have amnioserosa cells remaining at the midline, precluding normal interactions between contralateral CBs (Figure 5B–B’). In many embryos, development is disrupted to such a point that identification of the CBs is impossible (e.g., Figure 5B’). While dorsal closure succeeds, the layer of ectoderm is often thin and variable (Figure 5B–B’’). However, despite these defects in the surrounding tissues, in regions of the embryo where contralateral CBs meet at the midline, a lumen, albeit small, often forms (Figure 5B’’). Taken together with the tissue-specific dominant-negative data, this suggests that Src42A is not required in the CBs to promote lumen formation. Rather, the heart defects observed in *src42A* mutants are due to disruptions in dorsal closure and amnioserosa internalization. It also remains possible that Src42A has a heart autonomous role in subsequent lumen expansion, a process regulated by different genetic factors than initial lumen formation [61,62]. 

## 4. Discussion

CHDs encompass a variety of diseases that range in severity and treatment potential. Correctly and quickly identifying the disease and its etiology aids in determining an appropriate course of action. Knowledge of the causative factor is also important to prevent familial CHDs in pets such as dogs and cats that undergo extensive inbreeding [63]. Despite increasing recognition of the importance of understanding the genetic etiology, clinical studies are limited by the complexity and multifactorial nature of many CHDs. The experiments described here employ the genetic and cell biological strengths of the *Drosophila* model to explore the mechanism of heart development.

### 4.1. Genetic Analysis to Map Protein Networks Underlying Heart Development

As demonstrated by our lab and others (reviewed in [25]), the *Drosophila* heart is a helpful and established model organism to test the requirement and role of specific genes. For almost all genes in the fly genome, multiple mutant alleles are readily available through stock centers (e.g., the Bloomington Drosophila Stock Center) or other experimental research labs. Using three independently derived mutant strains, we demonstrated that a reduction or loss of Src42A results in an inability of the heart to properly form as the cells do not migrate fully to their necessary positions. The ability to test independently derived mutations gives confidence that the heart defect is directly caused by the loss of Src42A, and not by another unknown genetic abnormality.

Since a defective gene impacts the whole organism, the *src42A* mutant analysis does not reveal whether or not the heart defect is due to a requirement for Src42A in the heart itself or if it is a secondary defect due to Src42A’s role in surrounding tissues. Using the UAS-Gal4 system [55], cell or tissue autonomous roles can be directly tested by expressing transgenes in a temporally and spatially restricted manner. Since neither expression of normal Src42A nor a dominant negative version disrupts heart cell migration, it appears that the requirement of Src42A for heart development is due to Src42A expressed in non-heart tissues. However, since the dominant negative construct only disrupts the kinase domain of Src42A, it remains possible that the other domains of Src42A are required in the heart.

The ready availability of known mutations and the short life-cycle of flies also permits complex breeding schemes to test multiple genes together [64]. The term “genetic interaction” is used to describe mutations in two or more genes that are able to increase or decrease the severity of the phenotype relative to an additive effect of two independently acting genes. A genetic interaction suggests that the product of the two genes work together and such evidence can be used to map complex protein networks. Our combined analysis of *src42A* and *fak56D* suggests that Src42A impacts the heart in a Fak56D-independent manner. This is intriguing, as many studies in mammalian systems suggest that Src kinases and FAKs most commonly work as a complex [31,32,33]. 

### 4.2. Detailed Cell Biological Analysis Suggests that Src42A Functions in An Integrin-Independent manner

Both *Drosophila* and rodent (mice) models have demonstrated a requirement for the Integrin adhesion receptor during heart formation [16,17,27], but the complex nature of the mammalian heart precludes detailed analysis when development goes awry. We tested the hypothesis that Integrin signals through Src42A to control formation of the *Drosophila* heart tube. Several observations suggest that Src42A’s role in heart development is independent of Integrin. First of all, although heart development is disrupted in mutants for *src42A*, *scb* or *mys* (the genes for the *αPS3* and *βPS1* integrin subunits), and *rhea* (the gene that encodes Talin, a key Integrin signaling adaptor), the defects in *src42A* mutants were markedly different. In embryos with a genetic abnormality in *src42A*, the posterior cardial cells were frequently unable to reach the midline. This correlated with a posterior reduction in membrane dynamics as visualized in live embryos. In contrast, delayed cell migration in *scb*, *mys*, and *rhea* mutants was most frequently seen anteriorly [27,29] and the membrane dynamics were reduced along the entire length of the heart fields [27,29]. Furthermore, when cells in *src42A* mutants did reach the midline, they were able to form an open heart tube, albeit small. In contrast, the hearts in *scb*, *mys*, and *rhea* mutants remained closed [27,29]. Taken together, the detailed cellular analysis of the mutant embryos suggests that Src42A and Integrin-Talin signaling regulate different aspects of cardiac development. 

### 4.3. A Role for Transient Tissues in Proper Heart Development

Since the tissue-specific expression of Src42A-DN suggests that Src42A kinase is not required in the heart tissue itself, how then is Src42A required for heart development? In mammals, proper blood circulation requires closure of the ductus arteriosus, the vessel that allows blood to bypass the lungs pre-birth. Patent ductus arteriorsus, a failure to close off this blood vessel, leads to increased stress on the heart and lungs and possible congestive heart failure [65,66,67]. A study in swine demonstrated that proper ductus arteriorsus closure involves apoptosis or necrosis of cells within the inner layers of the vessel that are only required in the swine fetus [68]. Similarly, during the final stages of Drosophila embryonic heart development, the transient extraembryonic amnioserosa tissue internalizes and undergoes apoptosis [47]. However, careful analysis of development reveals that removal of the amnioserosa tissue only partially completes in src42A mutants. Rather, the amnioserosa cells persist at the intended site of heart tube formation and trigger secondary heart teratologies, including cardia bifida.
